# Correspondence Analysis to Demonstrate the Relationship Between Musculoskeletal Pain and Body Mass Index

**DOI:** 10.7759/cureus.40570

**Published:** 2023-06-17

**Authors:** Mili Sengar, Abhishek Gupta, Rajesh Kunwar

**Affiliations:** 1 Department of Community Medicine, TS Misra Medical College, Lucknow, IND; 2 Department of Community Medicine, Government Medical College, Kannauj, Kannauj, IND

**Keywords:** musculoskeletal pain, obese, overweight, correspondence analysis, biplot, dimension, inertia

## Abstract

Background

As obese people frequently experience persistent musculoskeletal pain (MSP), understanding the relationship between obesity and pain may help develop therapeutic and preventative approaches to treat discomfort from MSP. MSP can negatively impact such individuals’ quality of life and their ability to perform daily tasks. Therefore, more thorough investigations are required to fully understand the connection between obesity and MSP.

Aims

To assess the relationship between musculoskeletal pain(MSP) and body mass index (BMI) among women in the age group of 25 to 65 years.

Methods

From July to December 2022, a cross-sectional study among women between the ages of 25 and 65 was carried out near the Rural Health Training Centre (RHTC) of a medical college in the Lucknow District of Uttar Pradesh, India. In total, 443 women took part in the study. BMI was computed, weight and height were recorded, and MSP at any site (i.e., neck, shoulders, upper back, upper arms, lower back, forearms, wrists, hip/buttocks, thighs, knees, lower legs, and ankles) was noted. The data were analyzed through correspondence analysis.

Results

Of the 443 women that participated in the study, 224 (50.6%) had MSP, and 9.3% and 28.2% were obese or overweight, respectively. Obese and overweight women were found to be at a higher risk of upper and lower back pain.

Conclusion

In our study, a significant relationship between MSP and BMI was confirmed and visualized by correspondence analysis.

## Introduction

Musculoskeletal disorders are a prevalent source of pain and disability globally [1]. Compared to any other group of illnesses, musculoskeletal disorders significantly restrict functional abilities in the adult population. Worldwide, musculoskeletal pain (MSP) has a significant impact on years lived with disability in those affected [2].

Body shape and size influence susceptibility and resistance to disease [3]. Women’s lifestyles and aging are arguably more stressful and complicated compared with those of men because of hormone changes during menopause [4]. After menopause, women are more prone to various health problems such as cardiovascular diseases and osteoporosis [5]. Body mass index (BMI) is the most commonly used anthropometric method to assess obesity and related health risk. The BMI method classifies individuals into specific weight status categories that are associated with different levels of health risk [6].

Correspondence analysis (CA) is a technique that is specifically designed for the analysis of categorical variables and is used to depict relationships between the variables’ categories graphically [7]. Any forms of categorical variables (i.e., binary, ordinal, or nominal) can be analyzed because no distributional assumptions are needed. The usual purpose of using CA is to graphically represent the relative frequencies in terms of the distance between individual row and column profiles and the distance to the average row and column profile, respectively, by dimension reduction [8].

This study was undertaken to demonstrate a correspondence analysis (CA) between musculoskeletal pain (MSP) and body mass index (BMI).

## Materials and methods

From July to December 2022, a cross-sectional study among women between the ages of 25 and 65 was carried out in the catchment of a medical college’s Rural Health Training Centre (RHTC) in the Lucknow District of Uttar Pradesh, India. A sample size of 330 was computed using an absolute precision of 5% and a prevalence rate of 31.3% for MSP among women [9]. In total, 443 women took part in the study. Multistage sampling was chosen as the sampling strategy. The sample was obtained using a multistage sampling method. The first stage involved selecting two Subcenters out of four Subcenters in the catchment area of the RHTC (Rural Health and Training Centre) by simple random sampling. In the second stage, four villages were selected randomly from each Subcenter. In the third stage, population proportionate sampling was used to select participants from each village. The first household was selected randomly from each village, then a random table was used to select further households. One female of the age group 25-65 years was selected randomly from each household.

Data on the study participants were obtained by using a pretested questionnaire that asked each respondent for personal, socioeconomic, demographic, and geographic information. Any pain that had affected a muscle, bone, joint, ligament, or nerve over the past seven days was classified as MSP. The locations of the pain were also identified, which included the neck, shoulders, upper back, upper arms, lower back, forearms, hand/wrist hip/buttocks, thighs, knees, lower legs, and ankles [10]. When analyzing the findings, the most prevalent site of pain based on pain severity on a Likert scale was taken into consideration. To rule out any obvious medical conditions, the study participants received a general clinical examination. Height was determined using a stadiometer, and weight was determined using a weighing machine with an accuracy of 100 g while wearing minimal clothing. According to the WHO classification, the participants’ BMIs were divided into four categories: underweight, normal, overweight, and obese [11]. Underweight was defined as a BMI of 18.5 or less, normal as 18.5-24.9, overweight as 25-29.9, and obese as 30 or more.

Statistical methods

Stata software (StataCorp, College Station, Texas) was used for data analysis. CA was applied, and p-values of <0.05 were considered significant.

CA is a non-linear, multidimensional technique of multivariate descriptive analysis. The results obtained by CA can be seen not only analytically but also visually. The following terms were used while interpreting the CA results [7]:

1. Active margin: Summation of frequencies of rows and columns.

2. Mass: Marginal relative frequency.

3. Inertia: In correspondence analysis, the total variance is referred to as “inertia” and is a measure of the individual’s deviation relative to the typical profile. The inertia is obtained by dividing the Pearson chi-squared statistic by the entire sample size.

4. Model: The collection of one or more independent variables and their predicted interactions to explain variation in a dependent variable (i.e., to predict the outcome based on values of a set of predictor variables) [12].

5. Dimension: The most significant departures from independence are caused by inertia. The highest deviation from independence or the quantity of explained inertia is Dimension 1, followed by Dimension 2, and so on. Dimensions are created by locating the axes that maximize explained inertia while simultaneously minimizing the distance between the profiles and the axis. The further away from the origin a response category is along a particular dimension, the greater its influence on that dimension [7].

## Results

The mean age of the women was 42.08±11.47 years (range 25-65 years). Ninety-seven percent (97%) of the women were Hindu, 60.8% belonged to a joint family, and 26.7% belonged to the lower middle class. Nine point three percent (9.3%) of the women were obese, and 28.2% were overweight. Table 1 displays the frequencies for MSP, BMI, and the active margin, which is the sum of the frequencies. Two hundred twenty-four (224; 43.6%) of the 443 women reported having MSP. It is shown in Table 1 that out of the total number of women suffering from MSP, 77 (34.38%) were overweight and 37 (16.52%) were obese. Further, it can be seen in Table 1 that out of 443 women (with or without MSP), 69 were underweight, 208 were normal, 125 were overweight and 41 were obese. Hence, MSP was present in 61.6% of overweight (77 overweight women had MSP out of a total of 125 overweight women) and 90.24% of obese women (37 obese women had MSP out of a total of 41 obese women). 

**Table 1:** Correspondence table of MSP location and BMI *BMI=body mass index; MSP=musculoskeletal pain

MSP Location	BMI classification				
	Underweight (n=69)	Normal (n=208)	Overweight (n=125)	Obese (n=41)	Active Margin
Neck	0	3	0	6	9
Shoulder	2	1	8	0	11
Elbow	2	0	2	0	4
Hand/Wrist	1	0	0	0	1
Upper Back	0	4	2	6	12
Lower Back	14	31	43	19	107
Hip/Thigh	0	7	0	0	7
Knee	2	22	14	4	42
Ankle/Feet	1	20	8	2	31
Active Margin	22	88	77	37	224

The most important analytical table in CA is Table 2, which demonstrates the extreme statistical significance of this model (p<0.001).

**Table 2:** Summary table of correspondence analysis

Dimension	Singular Value	Inertia	Chi-Square	Significance	Proportion of Inertia		Confidence Singular Value	
					Accounted for	Cumulative	Standard Deviation	Correlation
1	0.453	0.205	146.896	<0.001	0.619	0.619	0.058	0.007
2	0.293	0.086			0.260	0.879	0.036	
3	0.200	0.040			0.121	1.000		
Total		0.332			1.000	1.000		

Additionally, it can be seen that CA produced three dimensions, used to describe our model (Table 2). Instead of producing all dimensions that could potentially explain something about the model, CA only produces those that can be comprehended. As a result, inertia does not equal 100%. The total variation explained by each model dimension is provided in the inertia column. The overall inertia (i.e., total variance explained) in the current study is 33.2%. Therefore, for our model, knowing something about MSP explains 33.2% of something about BMI and vice versa.

The amount of variance that the model accounts for in each dimension is listed. The most variance will always be explained by Dimension 1, then by Dimension 2, and so on. Table 2 shows that Dimension 1 explains 20.5% of the total 33.2% of variance accounted for, and Dimensions 2 and 3 explain 8.6% and 4% of it, respectively. The square roots of the inertia are shown in the “Singular Value” column. The percentage of variance that each dimension contributes to the total variance explained by the model is indicated by the values in the “Proportion of Inertia” column. Approximately 61.9% of the model’s total variance (33.2%) is explained by Dimension 1, 26.0% is explained by Dimension 2, and 12.1% is explained by Dimension 3.

Table 3 shows the "Overview Row Points" for the data, which provides details on how each row point is represented in the final biplot. The percentage of each MSP group in relation to the total number of MSP groups included in the analysis is shown in the table’s “Mass” column. The dimensions (1 and 2) where each row category will be located on the biplot are shown in the “Score in Dimension” column. The “Contribution” column shows how well each point fits into each dimension and how well the extraction of dimensions explains each point.MSP at the shoulder location contributed 19.1% in Dimension 1 but hardly contributed to Dimension 2 (0.2%). Additionally, Dimension 1 accounts for approximately 83.8% of the variance of shoulder MSP across BMI, whereas Dimension 2 accounts for approximately 0.7%.

**Table 3:** Overview row points *MSP=musculoskeletal pain

MSP Location	Mass	Score in Dimension		Inertia	Contribution				
		1	2		Of Point to Inertia of Dimension		Of Dimension to Inertia of Point		
					1	2	1	2	Total
Neck	0.040	−1.350	1.730	0.079	0.167	0.315	0.406	0.580	0.986
Shoulder	0.049	1.307	−0.129	0.044	0.191	0.002	0.838	0.007	0.845
Elbow	0.018	2.098	0.227	0.041	0.179	0.002	0.851	0.009	0.860
Hand/Wrist	0.004	2.998	0.607	0.041	0.091	0.004	0.430	0.015	0.445
Upper Back	0.054	−0.913	1.155	0.047	0.102	0.187	0.417	0.582	0.999
Lower Back	0.478	0.273	0.197	0.023	0.081	0.048	0.675	0.304	0.978
Hip/Thigh	0.031	−1.198	−1.404	0.048	0.102	0.161	0.408	0.488	0.896
Knee	0.188	−0.221	−0.444	0.019	0.021	0.096	0.216	0.755	0.971
Ankle/Feet	0.138	−0.459	−0.713	0.040	0.066	0.184	0.321	0.673	0.993
Active Total	1.000			0.382	1.000	1.000			

**Figure 1:**
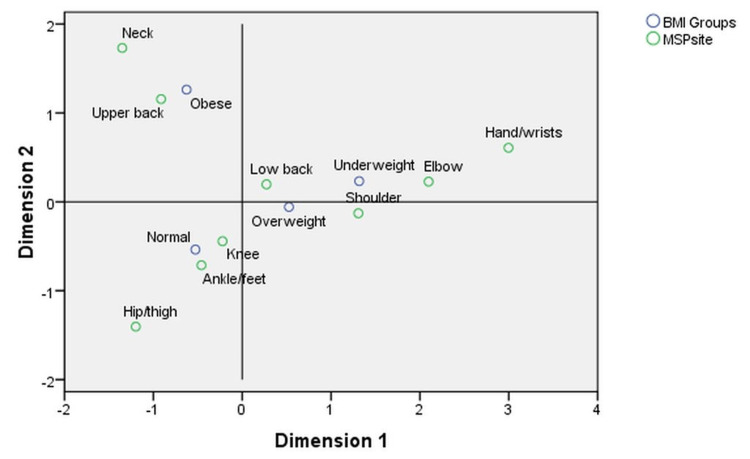
Biplot showing the relationship between MSP and BMI *MSP=musculoskeletal pain; BMI=body mass index

The “Overview Column Points” provides the same information for the plotting of column points on the biplot, as seen in Table 4, which shows that the obese group accounts more on Dimension 2 (~69%) and less heavily on Dimension 1 (14%). The extraction of Dimension 1 only accounts for approximately 22% of the variance in the obese group at all MSP locations, as opposed to that of Dimension 2, which accounts for approximately 78% of the variance. The horizontal axis in Figure 1 represents Dimension 1, and the vertical axis represents Dimension 2. Dimension 3 is not included in the biplot because it only explains 4% of the variance. As the neck is part of Dimension 1, which is farthest from the origin, it is of the greatest significance. The most significant MSP located along Dimension 2 is hand/wrist. These findings suggest that neck MSP showed the most significant difference or the largest divergence from independence. The distinction between hand/wrist and the other MSPs was the second-most significant one.

**Table 4:** Overview column points *BMI=body mass index; UW=underweight; N=normal; OW=overweight

BMI Group	Mass	Score in Dimension		Inertia	Contribution				
		1	2		Of Point to Inertia of Dimension		Of Dimension to Inertia of Point		
					1	2	1	2	Total
UW	0.098	1.317	0.232	0.098	0.388	0.014	0.762	0.021	0.782
N	0.393	−0.527	−0.537	0.094	0.248	0.296	0.510	0.461	0.971
OW	0.344	0.526	−0.059	0.061	0.217	0.003	0.687	0.007	0.694
Obese	0.165	−0.626	1.261	0.129	0.148	0.687	0.221	0.779	1.000
Active Total	1.000			0.382	1.000	1.000			

The chi-square statistic, which is based on the point distances of categories in a biplot, displays the strength of trends within data, whereas a measure of how different or similar two columns or row points are is provided by their distance from one another. In contrast to points that are mapped far apart from one another, those that are mapped near one another have comparable profiles. The biplot shown in Figure 1, which graphically portrays all of our data, exhibits some broad patterns, with the caveat that this specific model only accounts for 33.2% of MSP based on BMI. For example, obesity increases the risk of upper back pain, and being overweight increases that of lower back pain.

## Discussion

In the present study, the prevalence of MSP was found to be 50.6%, with lower-back pain being the most common site. Similar findings were found in a study conducted by Hendi et al. in which the prevalence of MSP was found to be 64.8%, with lower back pain being the most common location [13]. Similarly, 45% of women experienced MSP in a study conducted by Wijnhoven et al. [14]. A comprehensive review of MSP among nurses also showed that the lower back was the most common site of MSP [15].

The present study showed a positive relationship between MSP and high BMI. A study conducted by Rosa et al. showed that persistent MSK pain is a significant problem in obese patients [16]. Similarly, there was a high prevalence of MSP in individuals with severe obesity [17]. One meta-analysis revealed that overweight and obesity increase the risk of lower-back pain, and overweight and obesity had the strongest association with seeking care for lower back pain and chronic lower back pain [18].

CA allows for dimension reduction representation in the form of a graphical display, which allows for comparisons between variables, as well as between participants and variables in their relative placement in shared reduced dimensional space. The biplot analysis in Figure 1 showed that back pain was associated with high BMI. Similar findings were found in a study in which high-intensity lower back pain and/or disability were associated with increased levels of obesity [19].

Limitations of the study

There are two limitations to this study: 1) Both genders could be included in the study and then a comparison could be made between the two; 2) More body composition parameters like waist-hip ratio, body fat, visceral fat, etc. could have been studied in addition to BMI.

## Conclusions

The CA of the data provided an extremely useful general picture of associations between MSP and BMI. The graphical depiction of the different variables clearly outlined the relationship between back pain and high BMI. As seen in our study, CA is a very useful tool to find relationships between categorical variables and to develop a hypothesis, and it can be easily implemented to analyze and effectively interpret a set of data.
